# Assessing Changes in Airflow and Energy Loss in a Progressive Tracheal Compression Before and After Surgical Correction

**DOI:** 10.1007/s10439-019-02410-1

**Published:** 2019-12-02

**Authors:** Qiwei Xiao, Raul Cetto, Denis J. Doorly, Alister J. Bates, Jan N. Rose, Charlotte McIntyre, Andrew Comerford, Gitta Madani, Neil S. Tolley, Robert Schroter

**Affiliations:** 1 Department of Aeronautics, Imperial College London, South Kensington Campus, London, SW7 2AZ UK; 2 Department of Otolaryngology and Head and Neck Surgery, Imperial College Healthcare, St. Mary’s Hospital, Praed St, London, W2 1NY UK; 3 Department of Clinical Radiology, Imperial College Healthcare, St. Mary’s Hospital, Praed St, London, W2 1NY UK; 4 Division of Pulmonary Medicine, Cincinnati Children’s Hospital Medical Center, 3333 Burnet Ave, Cincinnati, OH USA; 5 Department of Bioengineering, Imperial College London, South Kensington Campus, London, SW7 2AZ UK

**Keywords:** Tracheal Airflow, Flow Energy Loss, Airway Resistance, Compressed Trachea, Inflow Truncation, Airway CFD

## Abstract

The energy needed to drive airflow through the trachea normally constitutes a minor component of the work of breathing. However, with progressive tracheal compression, patient subjective symptoms can include severe breathing difficulties. Many patients suffer multiple respiratory co-morbidities and so it is important to assess compression effects when evaluating the need for surgery. This work describes the use of computational prediction to determine airflow resistance in compressed tracheal geometries reconstructed from a series of CT scans. Using energy flux analysis, the regions that contribute the most to airway resistance during inhalation are identified. The principal such region is where flow emerging from the zone of maximum constriction undergoes breakup and turbulent mixing. Secondary regions are also found below the tongue base and around the glottis, with overall airway resistance scaling nearly quadratically with flow rate. Since the anatomical extent of the imaged airway varied between scans—as commonly occurs with clinical data and when assessing reported differences between research studies—the effect of sub-glottic inflow truncation is considered. Analysis shows truncation alters the location of jet breakup and weakly influences the pattern of pressure recovery. Tests also show that placing a simple artificial glottis in the inflow to a truncated model can replicate patterns of energy loss in more extensive models, suggesting a means to assess sensitivity to domain truncation in tracheal airflow simulations.

**Electronic supplementary material:**

The online version of this article (10.1007/s10439-019-02410-1) contains supplementary material, which is available to authorized users.

## Introduction

The trachea in the normal human adult may be idealized as a tube of length 10–12 cm, with a diameter of about 2 cm. For a healthy subject the effort needed to drive airflow through the trachea represents a minor contribution to the total work of breathing.^5^,^20^ However, with either intrinsic (e.g., Wegner’s granulomatosis^9^) or extrinsic (e.g., retrosternal goitre) pathological tracheal compression, tracheal airway resistance may become severe.^7^

When evaluating compression severity, detailed geometric quantification can now be obtained *via* reconstruction of the 3D airway from the original CT datasets. This provides more precise geometric data than that derived from axial image evaluation, but the relation between geometry and flow resistance is subtle. This is recognised in CT-based assessment of coronary artery disease, where computational fluid dynamics (CFD) prediction of pressure significantly improves assessment.^23^ Applied to the airways, although some validation studies have been performed,^14^ the use of CFD is less extensive than for the coronaries.

Nevertheless the potential scope for application of CFD to the airways is large. For instance, it is possible to provide measures such as pressure loss, resistance and wall shear stress which relate directly to patient symptoms.^12^–^13^,^19^,^26^,^31^ Studies^16^,^24^,^33^,^39^ have applied computational predictions to virtually model the effects of geometry modification on surgical intervention, with Chen *et al.*^10^ pointing out the use of CFD to evaluate the effectiveness of tracheal surgery in particular. However the generally high Reynolds numbers of large airway flows makes the task of resolving airflow turbulence difficult. Few investigations of realistic airways have utilised higher fidelity larger eddy simulation (LES), let alone attempted direct numerical simulation, DNS.^3^,^6^

Moreover few studies have included models based on actual post-operative geometries as in Takeishi *et al.*,^35^ who apply flow modelling to compare flow pre- and post-tracheal surgery in infants. Our present, imperfect knowledge of the healing process, among other factors, means actual post-operative geometries may differ considerably from those intended. Indeed surgery for enlargement of tracheal calibre might unintentionally induce losses due to separation.^35^ The long spatial extent of the upper airway introduces another complicating factor when dealing with clinical data: only part of the airway above the constriction might be included in the region imaged. Previous works^11^,^19^,^30^,^38^ discuss the necessity of including the complete or partial airway above the larynx to fully model normal tracheal flow. However, for the severe pathological conditions in the study^21^ pre- and post-operative CFD modelling of a tracheal stenosis is described without explicit consideration of upstream disturbances. Regardless, the level of flow disturbances created in the airway superior to the trachea is not fully known even with complete airway imaging. Tracheal inflow during inspiration can vary with posture, breathing mode, and laryngeal aperture. These boundary conditions can vary within and between subjects. It is thus important to re-assess the significance of upstream flow disturbances on measures of clinical interest, to determine whether computational prediction could be incorporated without alteration to established imaging protocols.

In this study, virtual 3D models of a worsening tracheal compression are generated from CT scan data using image segmentation. Estimated inhalation resistance and energy loss at several time points pre- and post-operation are then determined using LES computations. Using extended realistic and artificial inflow geometries, the present study investigates the scale of alterations in predictions of loss due to the neglect, or the variability of the upstream airway. Finally airway resistance predictions are compared to those of an idealised model study and the effectiveness of simple power law scaling demonstrated.

## Materials and Methods

### Image Data

The pathological trachea used for this study was from a patient with a retrosternal goitre amongst other co-morbidities, particularly chronic obstructive pulmonary disease (COPD).^20^ The subject underwent several CT scans for clinical reasons and the patient’s permission was obtained to analyze a series of those scans. Research and Ethics compliance was obtained from the local Joint Research Compliance Office Reference 15SM2566. The data was analyzed using current American Thyroid Association (ATA) imaging test guidelines^1^ for surgical intervention.

CT scans were obtained at quiet breathing with the patient in the supine position on three occasions before (T0,T4,T15) and one (T23) after surgical intervention; the intervening time in months between scans being prefixed T. The equipment used and slice details are given below.

- T0, (Phillips ICT 256), reconstructed as 1 mm thick slices;
- T4, (4 months after T0, Phillips Brilliance 64), reconstructed as 3 mm thick slices;
- T15, (15 months after T0, Phillips Brilliance 64), reconstructed as 2 mm thick slices;
- T23, (23 months after T0, Phillips Brilliance 16), reconstructed as 3 mm thick slices.

**Figure 1:**
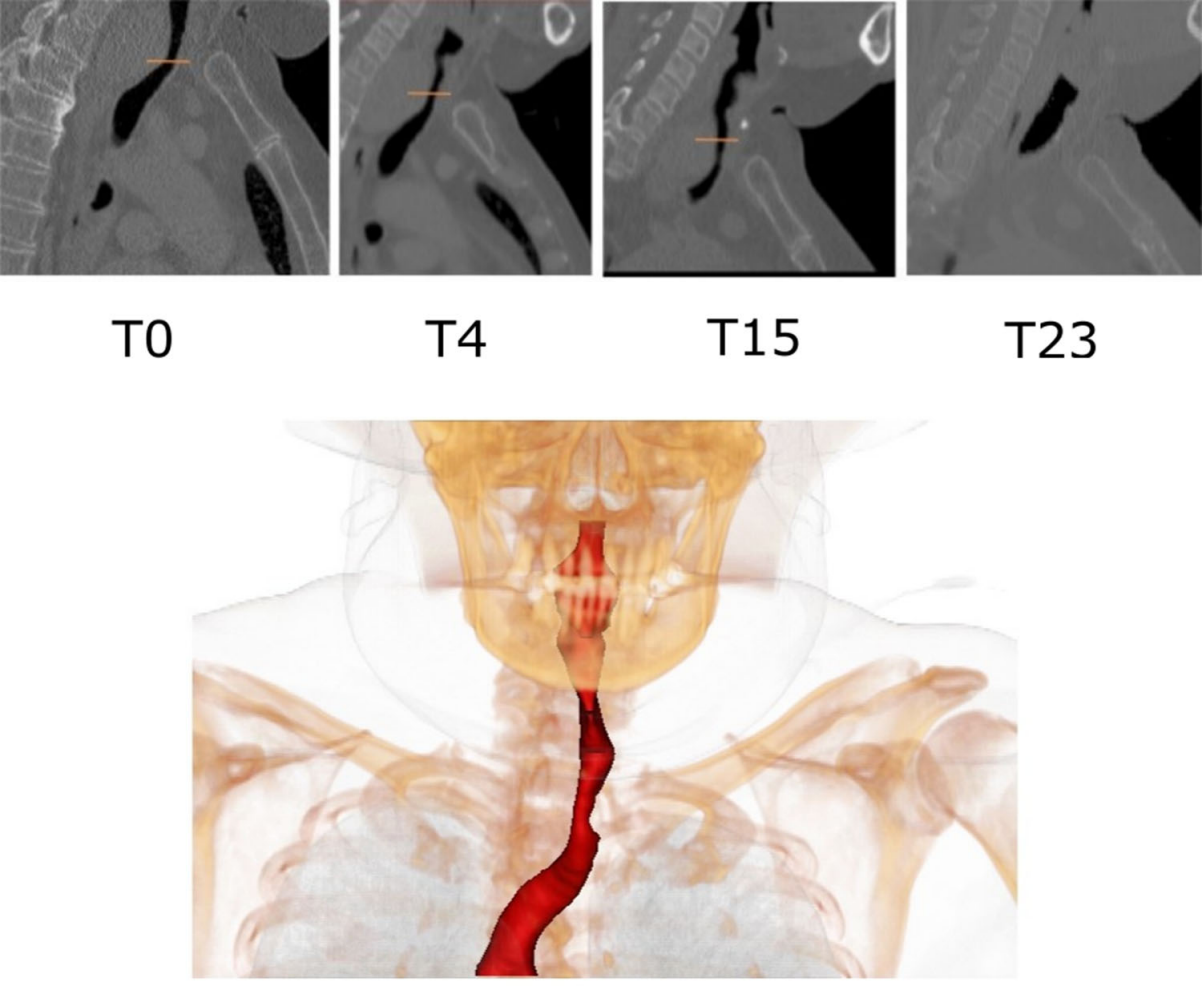
The top image is a sagittal view comparison corresponding to the point of maximal constriction in all scans. The yellow line in the picture indicates the location of minimum cross-sectional area (except T23). The lower image is a volume rendering of the subject’s anatomy at T15 showing the skeleton with a surface rendering of the segmented airway.

The in-plane resolution was 0.6 mm per voxel in all cases. The patient was symptom free at the time of scans T0 and T4, but exhibited respiratory symptoms two months before the last pre-operative scan at T15, reporting extreme difficulty taking every breath at T15. At the time of the final scan (T23), two months following total thyroidectomy, the patient reported total symptomatic relief. Figure 1 shows a sagittal view of one slice for all the CT scans with the yellow marker at the minimum cross-sectional position in the pre-operative scans.

### Airway Geometry and Simulation Definition

Airway lumens were reconstructed using threshold-based automated airway segmentations (pulmonology module^32^ of Mimics Innovation Suite: Materialise, Technologielaan 15-3001 Leuven, Belgium), as illustrated by the lower image in Fig. 1 for the T15 geometry. The length of the imaged airway varied depending on scan protocol: T0 includes part of the trachea (below first trachea ring and above carina) whereas the other studies included a greater portion of the airway, either to the nasopharynx or to just below the carina, but not simultaneously including both. A trachea centerline was generated from the reconstructed airway volume using the Vascular Modelling Toolkit (VMTK).^29^ An in-house MATLAB (R2016b, The MathWorks, Inc., Natick, MA, USA) code^3^ generated planes normal to the local centerline direction along the airway, allowing cross-sectional areas to be calculated.

Airway simulations have been found to match experimental results, with observed differences attributed to limitations of mesh resolution^2^,^17^,^25^ and model. From previous studies,^8^,^11^,^12^,^19^ LES as opposed to Reynolds-averaged Navier–Stokes (RANS) methods are preferable to capture details such as jet break up.^22^ Given a mean tracheal flow transit time of ≈ 25 ms and Womersley number < 1 for the modelled ventilation rate, a steady flow assumption is considered appropriate.^28^

The CFD software Star-CCM+ 11.04.012-r8 (Siemens Product Lifecycle Management Software Inc, Plano, TX, USA 75024) was used for mesh generation and to solve the Navier–Stokes equations. Quasi-steady, i.e. unsteady simulations with constant prescribed velocity inflow, were performed mostly for a flow rate of 392 mL s^−1^ (23.5 L min^−1^). This corresponds to the peak value of an inhalation waveform matching the first half of a sinusoid of period 4 s, with 500 ml inhaled volume. (It is also equivalent to constant 61 min^−1^ ventilation at a breathing rate of 12 min^−1^ and inspiratory-expiratory ratio of 1:3. Flowrates from 200 to 833 mL s^−1^ were also computed on the Imperial College Research Computing Service.^18^

Large Eddy Simulation (LES) with the WALE sub-grid scale model was used for flow prediction, as previously described.^4^ Meshes ranged from 3.5 to 5 million polyhedral elements, including wall prism layers; average first layer height ranged from 0.013 to 0.023 mm, time step was 5 × 10^−5^ s. Inlet and outlet extensions were added to the imaged geometry by extruding the end- airway cross-section by 35 mm (around 3 hydraulic diameters) at all boundaries. The pressure outlet boundary condition was specified in Star-CCM+, corresponding to prescribed static pressure with zero order extrapolation of velocity components. Run times were 0.2 s physical time, with the first 0.05 s discarded to allow start-up transients to clear. Mean tracheal pressure loss computed over the intervals [0.05 s, 0.125 s] and [0.125 s, 0.2 s] differed by only approximately 0.5%, so averaging over [0.05 s, 0.2 s] was deemed adequate. Further computational details are given in the supplementary material.

## Results

### Geometry

**Figure 2:**
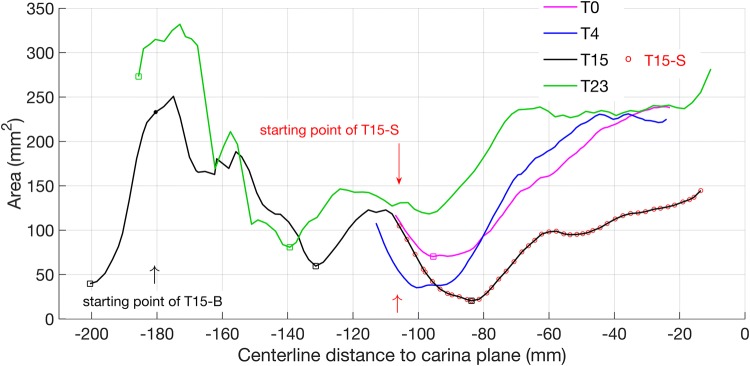
Line plots of cross-sectional area distribution for all geometries. The three square markers on the curve for T15 correspond to cross sectional area minima: from left to right of x-axis these minima are at the base of tongue (≈ *x* = − 200, glottis, *x* ≈ − 135 and overall minimum area *x* ≈ − 85). In addition, the circles overlapped with the T15 curve represent the extent of a truncated T15 geometry created to compare with that at T4. Geometry T23 does not extend superiorly quite as far as T15 and has minimum area at the glottis, square marker at ≈ − 140.

Cross-sectional area distributions for the virtual airway models derived from the subject imaged at different time points are shown in Fig. 2, where the most negative location corresponds to the most superior position. Anatomical landmarks in the image data were used to translate area distributions (approximately, given the progressive airway distortion) to an estimated common carina origin. The area distribution curve for the T15 model shows three pronounced minima, highlighted by larger markers. From left to right these correspond to: (i) base of the tongue (associated with partial airway collapse as the subject was imaged in the supine position), (ii) glottis, (iii) the point of maximal tracheal compression. Partial airway collapse in the tongue base region was also noted in a previous study.^6^

For the data set considered here, only a sub-glottal part of the trachea is common to both of the later pre-operative scans. The scan data for T0 extends from above the carina to approximately the first tracheal ring. The T15 geometry also ends an estimated 9 mm above the carina, though includes the supra-glottic airway. The geometries from scans at T0, T4 and T23 extend to the carina. However, the area distribution plot does not extend as far: the procedure to define a conduit centreline terminates before a branch.

The length of the constricted portion was defined as a contiguous segment with lumen cross-sectional area (*CSA*) reduced by 35% or more^34^ compared to the first tracheal ring area, Table 1.

**Table 1:** Geometric data of tracheal lumens.

CT scans	Constriction length (mm)	Min. cross-sectional area, *A*_C_ (mm^2^)	Glottis area (mm^2^)	First trachea ring area (mm^2^)
T0	17.0	70.3	Not available	Not available
T4	32.0	35.2	Not available	148
T15	32.7	20.5	66.9	117
T23	0	80.8	80.8	146

The constriction ratio is defined as the *CSA* ratio: $(CSA_{\text{First ring}} - CSA_{\text{min}})/CSA_{\text{First ring}}$ and is 82.5% for T15

From the table, in the 15 months between scans T0 and T15, the minimum cross-sectional area (${CSA}_{\text{min}}$ or $A_{\text{C}}$) reduced from 70.3 to 20.5 mm^2^, whilst the constriction length was less altered. In the T15 geometry, $A_{\text{C}}$ is reduced by 82.5% relative to first ring area. Surprisingly the patient at T4 showed no breathing difficulty symptom, although the constriction ratio for T4 already reached 76%. The progressive change of compression that occurred after T4 corresponds to the significant breathing difficulty reported by the patient. After surgical correction, the tracheal airway ${CSA}_{\text{min}}$ reverted to its normal location at the glottis.

**Figure 3:**
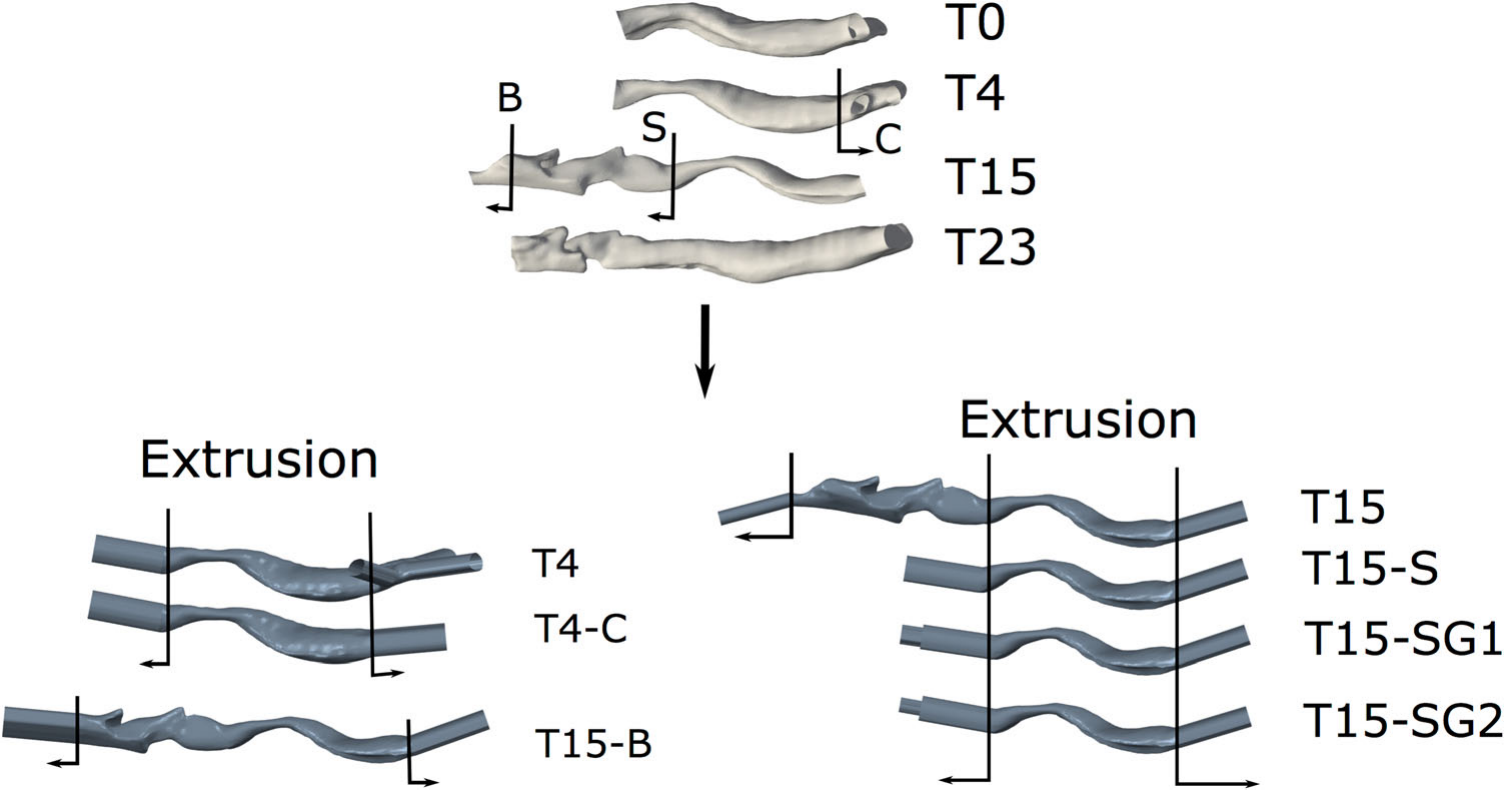
Upper: reconstructed airways at different time points in supine orientation. Lower: models used for flow prediction incorporate extruded cross-sections (35 mm in length) at inflow/outflow boundaries as indicated. The series of models shown is derived by truncation to assess scale of effect of neglected or variable geometry on losses attributable to constriction.

Relating the change in the imaged geometry to the change in airway resistance is subject to uncertainties, including due to differences in the imaged region and due to intra-subject variation. To provide equivalent bases for comparison and to estimate the scale of effects due to variation in flow, the imaged geometries were translated to a series of models (Fig. 3) as follows.

- Sub-glottal tracheal-only geometries: T15-S vs. T4. The part of T15 inferior to the first ring is created as a separate model,T15-S, by truncating the superior airway to marker “S” in Fig. 3, to compare with T4. Also model T4-C ( supra-carina outflow truncation) is made for comparison with T4 (to check outlet boundary condition influence).
- Complete laryngo-tracheal geometries: Model T15 (entire region) vs. post operative geometry T23. Model T15-B created by truncating T15 below tongue base constriction for comparability with T23. (Model T4-L created by grafting superior T15 airway on to T4 is discussed in supplementary information).
- Sub-glottal trachea-only models T15-SG1, -SG2 are similar to T15-S, but each incorporates an artificial constriction in its extruded inlet to generate inflow disturbances.

### Flow Analysis

Air flowing through the airways loses energy due to viscous dissipation, which results in the airway resistance component of the work of breathing. Flow losses are commonly estimated from a combined application of pipe friction correlations and loss factor; previously an idealised tracheal stenosis was characterised in terms of an appropriate loss factor.^7^

Pedley^28^ presents an analysis of energy loss and provides expressions for idealised flows, whilst the general relation between work-energy balance and pressure is described in many sources.^15^ For the case of a rigid duct, and neglecting the contribution of boundary shear work, the energy balance simplifies to:

1 $$\begin{aligned} \frac{\partial }{\partial t}\int _{V} \frac{1}{2}\rho |\mathbf u |^2 {\text{d}}V + \int _{S_1\cup S_2}(\mathbf u \cdot \mathbf n ) [P_s + \frac{1}{2}\rho |\mathbf u |^2 ]{\text{d}}S = \int _{V} \Phi {\text{d}}V \end{aligned}$$

where *V* is the volume of the duct between inflow and outflow plane $S_1$ and $S_2$ respectively, $\Phi$ represents the dissipation function, $\Phi =\tau : \nabla \mathbf u$ with $\tau$ the viscous stress tensor. Time-averaging removes the first (fluctuating kinetic energy) term, and thus for steady inflow, the temporal mean of the accumulated energy loss can be accounted for at any point by the difference in the mean value of total energy flux at the given station, say *i*:

2 $$\begin{aligned} E_{F,i} =\int _{S}\overline{(\mathbf u \cdot \mathbf n )[P_s+\tfrac{1}{2} \rho |\mathbf u |^2]} {\text{d}}S \end{aligned}$$

and that at inflow. The mean energy flux $E_{F,i}$ is approximated by integration of the product of time-averaged total pressure and normal velocity over the plane *i*. The slope at a point on a plot of $E_F$ against distance from inflow indicates the mean local rate of dissipation. Energy dissipation can be considered to comprise two parts: that required to overcome wall friction, and an excess due to flow non-uniformity.^5^ Where flow encounters a localised constriction, dissipation at first rises through increased wall friction; downstream, where the geometry typically expands, a strongly non-uniform jet flow is produced that dissipates through internal shear layers, further increasing losses. Likewise, where the trachea is significantly deviated, the turning required of the flow introduces bias, augmenting losses.

#### Sub-Glottal Trachea Models: Effects of Constriction on Flow

**Figure 4:**
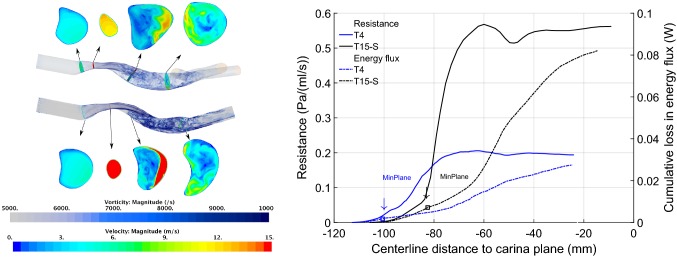
Left: comparison of vorticity and velocity for geometries T4 and T15-S. Cross-sectional planes indicate velocity distribution; volume rendering reveals vorticity magnitude, highlighting regions of disturbed flow. Right: resistance and energy flux comparison for truncated tracheal geometries.

Removing the supra-glottal airway reduces computational effort; it also provides a no-disturbance benchmark to study disturbed inflows. Figure 4 compares the flow in the comparably truncated geometries T4 and T15-S. Volume renderings of vorticity magnitude indicate two noteworthy features of the respective flows: (i) higher downstream vorticity in the case of T15-S, signifying greater energy dissipation, and (ii) the flow jet emerging from the trachea does not begin to break up immediately in either geometry.

Truncation results in a nearly uniform inflow at the sub-glottal inflow (SGI) plane in both T4 and T15-S, (see velocity contours in Fig. 4). At the respective $CSA_{\text{min}}$ locations, both lumens are elliptical, with the smaller lumen of T15-S producing a stronger flow jet than in T4. Planes downstream of the constriction show a highly asymmetric velocity distribution in both T4 and T15-S. This is due to the impingement of the jet and complex geometric shape of the cross-section; such non-uniformity of flow may be expected to lead to enhanced losses.^5^

#### Sub-Glottal Trachea Models: Effects of Constriction on Resistance and Energy Loss

Figure 4, shows the resistance and cumulative loss in energy flux, plotted from the start of the imaged region. Resistance increases markedly from the T4 to the T15-S geometry with reduction in $CSA_{\text{min}}$. The apparent overshoot in resistance, where the lumen expands downstream of the compression, reflects the averaging of pressure across a larger area. The variation of the mean flow power loss as given by Eq. (2), right axis of the plot in Fig. 4, reveals that enhanced energy dissipation occurs over the region where the flow jet emerging from the constriction breaks up and mixes. Whereas mean flow power continuously reduces along the trachea, flow resistance may appear to fluctuate. The cumulative loss curve thus identifies regions of dissipation and provides a robust means to chart the constriction energy cost. Note that whereas geometry T4 extends to the bifurcation, T15-S does not. The distribution of loss in the trachea almost to the carina is not altered (see supplementary information) whether the bifurcation is present or is replaced by a single outflow, (model T4-C). Static changes in geometry seem to have limited upstream influence. However the constriction may impose additional losses further downstream; to account for these requires both further geometry and flow split data.

Overall, geometry T15-S has a resistance about 2.4 times that of T4; their resistance ratio scales with exponent $\simeq$ 1.9 or nearly quadratically with the reciprocal of the constriction area ratio, $(CSA_{{\text{min}},T4}/CSA_{{\text{min}},T15})^2 = 2.9$.

#### Complete Laryngotracheal Geometry: Constriction Effects on Flow

**Figure 5:**
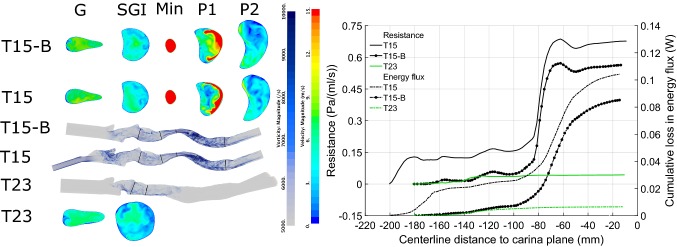
Comparison of geometry T15, T15-B and T23. Left: cross-sectional planes indicate velocity distribution; volume rendering reveals vorticity magnitude. Right: Resistance and energy flux for same geometries. For post-operative geometry T23, losses are small, with the glottis the most significant source. Patterns of loss at glottis and constriction for T15, T15-B are similar despite change in inflow.

Flow in the complete pre- and post-operative geometries (T15, T23) are compared in Fig. 5, along with the T15-B model. T15-B is created by truncating T15 just below the tongue base area contraction, providing a supraglottal inflow similar to model T23. Considering first the T15 geometry, flow exits from the narrow passage area (45 mm^2^) at the tongue-base as a stronger flow jet than that produced at the glottis, (area 67 mm^2^). Flow from the nasal, oral or combined oro-nasal airways passing through such a contraction would experience a pronounced acceleration, reducing the relative strength of disturbances arising from those parts of the airway geometry. This effect has been shown for a different complete airway geometry^6^; similar means to reduce inflow turbulence are used in wind tunnels.

The vorticity renderings of Fig. 5 show similar flow patterns in both T15 and T15-B geometries from the glottis downstream; also for the T23 geometry, the glottis is seen to be the main source of flow disturbances. From the velocity contours, the flow in T15 and T15-B downstream of the glottis is similarly disturbed at the start of the constriction, in marked contrast to the uniform inflow for T15-S, (previous Fig. 4). Comparing velocity contours in Figs. 4 and 5 shows that the jet exiting the constriction undergoes a more rapid break up for both T15 and T15-B than for the truncated T15-S geometry. This suggests an association between inflow disturbances and accelerated jet breakup.

#### Complete Laryngotracheal Geometry: Constriction Effects on Losses

For the T15 geometry, the flow jet emerging from the tongue base region produces an initial, rapid rise in resistance, whereas the energy flux deficit increases gradually. The loss profiles in this region are similar in shape to those associated with the constriction, though the latter are much larger. Model T15-B lacks the tongue base jet inflow, consequently it sees resistance and energy flux deficit rising only as flow accelerates through the glottis, (position $x \simeq -\,135$) and then mixes, for *x* in the range $[-\,140, -\,100]$. Beyond this point, the rate of loss accumulation and pattern of resistance appears almost exactly as for T15, with a simple offset equivalent to the tongue base loss. Further downstream (*x* in range $[-\,100 -\,20]$), the patterns of resistance and energy flux for both T15 and T15B are also similar to those for the truncated T15-S geometry.

The tracheal airway resistance at the prescribed flowrate of 23.5 L min^−1^ for T15 is 0.68 Pa mL s^−1^. At 30 L min^−1^ this would scale (using an exponent of 2, corresponding to a simple orifice plate as described further) to 1.16 Pa mL s^−1^, equivalent to 11.8 cm H_2_O L s^−1^. This is far above normal total airway resistance (let alone tracheal resistance), ≈ 1 cm H_2_O L s^−1^ at a 30 L min^−1^ flowrate.^20^ In the post-operative geometry by contrast, the flow resistance value of 0.05 Pa mL s^−1^ closely matches that of normal and idealised tracheas,^17^,^26^ adjusted for comparable flow rate. Moreover, resistance is now almost entirely attributable to the glottis. Results thus indicate an objective surgical benefit from the perspective of fluid dynamic efficiency.

#### Truncation Effects on Mechanics of Flow and Losses

**Figure 6:**
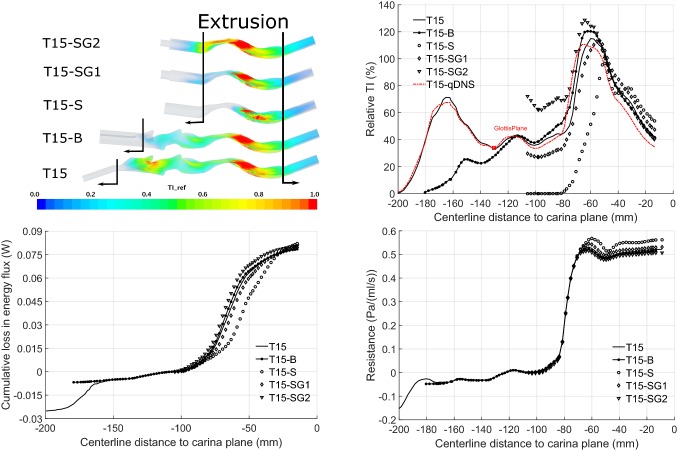
Volume renderings of turbulence intensity (upper, left), distribution of cross-section averaged turbulence intensity (upper right), energy flux (lower left) and resistance (lower right) for all T15 geometries.

The condition of flow directed through the larynx is generally uncertain, more so when imaging restrictions force inflow truncation. To investigate further, additional geometries labelled respectively T15-SG1 and T15-SG2 were created. For these geometries, the inflow extrusion of T15-S is modified to create a step expansion approximately three diameters upstream of the inflow, see Figs. 2 and 6. This simple and readily implemented artifice does not replicate the real glottic and supra-glottic airway. Instead it provides a convenient means to assess the influence of disturbances of different intensity (controlled by the constriction ratio and location).

**Table 2:** Geometric data for T15 and all T15-S cases.

Geometry	Glottis area (mm^2^)	First trachea ring area (mm^2^)	Inflow constriction ratio (%)
T15	66.9	117	43
T15-S	Not available	Not available	0

Geometry	Inlet area (mm^2^)	Inflow cross-sectional area (mm^2^)	Inflow constriction ratio (%)
T15-SG1	68.3	121	44
T15-SG2	38.2	121	68

Table 2 compares the inflow constriction ratios for original T15 and derived geometries: as indicated, the natural glottis constriction ratio (T15) is 43%, that for T15-SG1 is similar, while for T15-SG2, the constriction is more severe. Volume renderings in Fig. 6 (upper left) display the normalised turbulence intensity scalar. Label ‘TI-ref’ indicates turbulence intensity (supplementary information) is calculated with velocity at the first tracheal ring of model T15 as reference. Line plots in Fig. 6 (upper right) compare the variation in turbulence intensity for the different inflows. Results for a qDNS calculations are also included, showing generally good agreement with LES. From the plots of turbulence intensity, flow disturbances produced by T15-SG1 are of slightly lower intensity than those for T15 near the level of the first tracheal ring, despite similar glottis area. However reducing the modelled glottis area (model T15-SG2) enhances turbulence production, leading to inflow disturbances much above those in the T15 geometries.

When the results for different models are brought to a common zero at the start of the common geometry section, the distributions of energy loss and resistance (lower left and right of Fig. 6) show clear trends. For the energy loss, (lower left in Fig. 6) the onset of jet breakup is most delayed for the zero inflow disturbance model T15-S. It occurs increasingly early as disturbance level rises through T15-SG1, T15, and T15-SG2, matching the order in the respective turbulence levels, upper right of Fig. 6. This is despite differences in the manner of disturbance production in real versus extruded geometries. Similar effects are observed for the wall shear stress distribution (supplementary information). In quantitative terms, for the truncated T15-S geometry, the rise in energy loss is delayed by approximately 10 mm compared to the full geometry, mirroring the delay in jet breakdown observed in the vorticity renderings.

Although the rise in energy loss is delayed for the disturbance-free truncated geometry T15-S, this geometry shows an accumulated loss above that for all geometries with disturbed inflows. It appears that inflow disturbances promote earlier jet breakup, as found in a different context,^40^ and lead to slightly reduced energy losses due to the constriction. Correspondingly, overall tracheal resistance is affected by the level of upstream flow disturbances, lower right line plot in Fig. 6. For the range of disturbances considered, differences in the jet break up are evident, yet effects on resistance appear relatively small: resistance for the simple truncated case is only about 7% greater than for the more complete geometry. Similar effects are also found for the less severe constriction at T4, (supplementary information).

This is significant as it suggests the airway resistance of constrictions similar in form to those considered are largely unaltered by upstream geometry. Moreover it appears that a reasonably accurate estimate of the scale of the contribution of the neglected geometry may be derived by simple addition of an artificial orifice. These results provide a mechanistic underpinning to findings regarding the influence of upstream disturbances in a previous study,^11^ and invite comparison with findings in other contexts.^36^,^40^

#### Power Law Fit of Pressure Flow Relation

Both truncated and full geometries of the most constricted geometry T15 (82.5% constriction, were tested for a range of inspiratory flow rates (Table 3):

**Table 3:** Data for power law fit for flow rate vs. pressure loss of the most constricted geometry, T15.

	Flow rate (L min^−1^)	23.5	30	40	50
A	T15: Pressure loss inlet to outlet $P_{\text{inlet}} - P_{\text{outlet}}$ (Pa)	276.8	446.5	774.5	1223.9
B	T15: Pressure loss first ring to outlet $P_{\text{plane SGI}} - P_{\text{outlet}}$ (Pa)	206.8	331.9	582.3	911.3
C	T15-S: Pressure loss first ring to outlet $P_{\text{plane SGI}} - P_{\text{outlet}}$ (Pa)	223.6	357.6	616.3	950.9

Row A: whole airway losses; B: losses for whole airway measured from first ring; C: losses for truncated airway geometry (same extent as B)

Row B of Table 3 shows the total pressure loss of T15 within the common geometry section shared with T15-S, i.e. between plane SGI and outlet. We assume pressure loss across the constriction *Q* to be related to flowrate by a simple orifice-type power law

3 $$\begin{aligned} \Delta P = \tfrac{1}{2} \rho K \left( \frac{Q}{A_{C}}\right) ^n. \end{aligned}$$

Brouns *et al.*^7^ modelled tracheal stenoses of varying degrees in an idealised airway model. They propose taking *K* = 1.2 as a reasonable fit for a range of stenosis severity; *K* = 1.2 agrees with that expected for the present constriction from engineering correlations (supplementary material). With (*K*, *n*) = (1.2, 2), Eq. (3) predicts a loss $\Delta P \simeq$ 280 Pa for the T15 geometry at a flowrate $Q = 23.5 \, \hbox {L}\,\hbox {min}^{-1}$, almost the same as the overall tracheal loss for T15 (276.8 Pa, Table 3). However, the present geometries show proportionately much higher losses in the supraglottic airway than for the idealised airway. Removing the supraglottal contribution, the comparison is not as good: (i) considering just the subglottic loss for T15, over the range of flowrates, the prediction^7^ of $\Delta P$ is nearly 30% too high; (ii) with the disturbance free inflow of T15-S, the pressure loss is over-predicted by between 15 and 23% from lowest to highest flow rates. Similar findings hold for the T4 geometries (supplementary information).

**Fig. 7:**
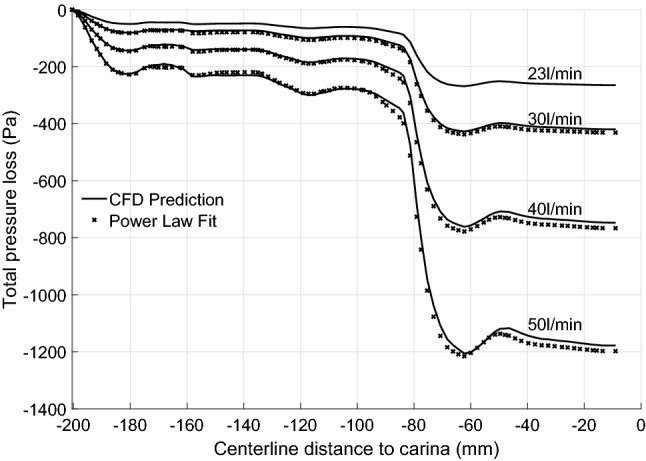
Power law predictions of T15 geometry with four different flow rates.

Equation 3 may not be capable of highly accurate loss estimation for a common *K*. However the increase in loss with increasing flowrate is predicted with surprising accuracy using a quadratic exponent, Fig. 7. There the pressure loss distributions for the lowest flow rate (23.5 L min^−1^) are scaled for higher flowrates by the square of the flowrate ratio; there is remarkable agreement of scaled and directly computed values. In terms of Eq. (3), for *n* =  2, choosing *K* =  0.95 gives an error of only 2% for $\Delta P$ across all flowrates.

## Discussion

Routinely, to diagnose respiratory pathology, spirometric testing is undertaken. Total airway resistance (between mouth and alveoli) can be measured in the clinical setting using very long established methods, but this requires a degree of patient cooperation that sometimes cannot be achieved in the most compromised cases. The traditionally measured flow volume loop (FVL), provides helpful insight into a wide variety of respiratory disorders.^27^ However, although FVLs are very widely used, they have various important limitations. For instance, the inspiratory limb is effort dependent and FVLs yield only global information regarding airways and parenchyma - they cannot give localized information or quantities in the way that CFD predicts. The approach proposed in the present study complements spirometry by providing mechanistic insight into the effects of an extrinsic compression on breathing mechanics. Firstly, the airway morphology can be quantified by volumetric reconstruction. Secondly, using CFD, the distribution of resistance and energy loss throughout the length of the trachea can be derived, providing an objective measure of the effect of a progressive extrinsic compression.

A common issue to be addressed in applying CFD to patient image data is that of inflow domain truncation. Capturing the supra-glottic airway allows its contribution to airway resistance to be included, but it may not be imaged due to other clinical priorities. In this work, inflow truncation was shown both to remove flow disturbances due to shear layer breakdown in the supra-glottic airway and to delay the breakdown of the jet issuing from the tracheal contraction. It was also found (supplementary materials) to affect the wall shear stress distribution and static pressure recovery. The effect of inflow disturbances is of broad interest.^40^ Although the present results point to differences in flow behaviour that may prove significant for applications such as aerosol delivery, inflow truncation was found to have relatively minor (less than 10%) effect on predicted flow loss due to tracheal compression.

The major contribution to energy dissipation and airway resistance was found to be associated with the region of minimum area. Since predictions of subglottic losses differ significantly from an idealised model, factors other than supra-glottic losses, such as the degree of lumen and axis distortion, may be significant. Nevertheless, it was found that losses scaled with reduction in cross-sectional area and with flow rate nearly as a simple quadratic power law, similar to the scaling observed in the idealised model of Brouns *et al.*^7^ Practically, this can reduce the amount of computational effort, replacing simulations to determine loss at each flow rate of interest by scaling where feasible.

Finally the present model is limited both in terms of the neglect of the potential for further downstream losses as already discussed and by the assumption of a rigid airway. The effect of the different pressure loadings on the tracheal wall during inspiration versus expiration may produce very different cross-sectional shapes and thus values for tracheal resistance, analogous to those seen in upper airway collapse.^37^

## Electronic supplementary material

Below is the link to the electronic supplementary material.

Electronic supplementary material 1 (PDF 3531 kb)

## References

[1] Alexander EK, Pearce EN, Brent GA, Brown RS, Chen H, Dosiou C, Grobman WA, Laurberg P, Lazarus JH, Mandel SJ (2017). 2017 guidelines of the american thyroid association for the diagnosis and management of thyroid disease during pregnancy and the postpartum. Thyroid.

[2] Ball C, Uddin M, Pollard A (2008). High resolution turbulence modelling of airflow in an idealised human extra-thoracic airway. Computers & Fluids.

[3] Bates A, Cetto R, Doorly D, Schroter R, Tolley N, Comerford A (2016). The effects of curvature and constriction on airflow and energy loss in pathological tracheas. Respiratory physiology & neurobiology.

[4] Bates A, Comerford A, Cetto R, Doorly D, Schroter R, Tolley N (2017). Computational fluid dynamics benchmark dataset of airflow in tracheas. Data in Brief.

[5] Bates A, Comerford A, Cetto R, Schroter R, Tolley N, Doorly D (2016). Power loss mechanisms in pathological tracheas. Journal of biomechanics.

[6] Bates AJ, Doorly DJ, Cetto R, Calmet H, Gambaruto A, Tolley N, Houzeaux G, Schroter R (2015). Dynamics of airflow in a short inhalation. Journal of the Royal Society Interface.

[7] Brouns M, Jayaraju ST, Lacor C, De Mey J, Noppen M, Vincken W, Verbanck S (2007). Tracheal stenosis: a flow dynamics study. Journal of Applied Physiology.

[8] Calmet H, Gambaruto AM, Bates AJ, Vázquez M, Houzeaux G, Doorly DJ (2016). Large-scale cfd simulations of the transitional and turbulent regime for the large human airways during rapid inhalation. Computers in biology and medicine.

[9] Cebral JR, Summers RM (2004). Tracheal and central bronchial aerodynamics using virtual bronchoscopy and computational fluid dynamics. IEEE transactions on medical imaging.

[10] Chen F-L, Horng T-L, Shih T-C (2014). Simulation analysis of airflow alteration in the trachea following the vascular ring surgery based on ct images using the computational fluid dynamics method. Journal of X-ray science and technology.

[11] Choi J, Tawhai MH, Hoffman EA, Lin C-L (2009). On intra-and intersubject variabilities of airflow in the human lungs. Physics of Fluids.

[12] Comerford A, Gravemeier V, Wall W (2013). An algebraic variational multiscale–multigrid method for large-eddy simulation of turbulent pulsatile flows in complex geometries with detailed insight into pulmonary airway flow. International Journal for Numerical Methods in Fluids.

[13] Cui X, Gutheil E (2011). Large eddy simulation of the unsteady flow-field in an idealized human mouth–throat configuration. Journal of biomechanics.

[14] de Rochefort L, Vial L, Fodil R, Maitre X, Louis B, Isabey D, Caillibotte G, Thiriet M, Bittoun J, Durand E (2007). In vitro validation of computational fluid dynamic simulation in human proximal airways with hyperpolarized 3he magnetic resonance phase-contrast velocimetry. Journal of applied physiology.

[15] Donati F, Figueroa CA, Smith NP, Lamata P, Nordsletten DA (2015). Non-invasive pressure difference estimation from pc-mri using the work-energy equation. Medical image analysis.

[16] Frank-Ito DO, Kimbell JS, Laud P, Garcia GJ, Rhee JS (2014). Predicting postsurgery nasal physiology with computational modeling: current challenges and limitations. Otolaryngology-Head and Neck Surgery.

[17] Heenan A, Matida E, Pollard A, Finlay W (2003). Experimental measurements and computational modeling of the flow field in an idealized human oropharynx. Experiments in Fluids.

[18] Imperial College Research Computing Service. 10.14469/hpc/2232..

[19] Lin C-L, Tawhai MH, McLennan G, Hoffman EA (2007). Characteristics of the turbulent laryngeal jet and its effect on airflow in the human intra-thoracic airways. Respiratory physiology & neurobiology.

[20] Loring SH, Garcia-Jacques M, Malhotra A (2009). Pulmonary characteristics in copd and mechanisms of increased work of breathing. Journal of applied physiology.

[21] Mason EC, Mcghee S, Zhao K, Chiang T, Matrka L (2019). The application of computational fluid dynamics in the evaluation of laryngotracheal pathology. Annals of Otology, Rhinology & Laryngology.

[22] Mihăescu, M., E. J. Gutmark, R. Elluru, and J. P. Willging. Large eddy simulation of the flow in a pediatric airway with subglottic stenosis. 47th AIAA Aerospace Sciences Meeting including The New Horizons Forum and Aerospace Exposition , 2009.

[23] Morris, P. D, D. Silva Soto, J. Feher, D. Rafiroiu, A. Lungu, S. Varma, P. Lawford, D. Hose, and G. JP. Fast virtual fractional flow reserve based upon steady-state computational fluid dynamics analysis: Results from the virtu-fast study. JACC Basic Transl. Sci. 2:434–446, 2017. 10.1016/j.jacbts.2017.04.003 PMC5582193 28920099

[24] Mylavarapu G, Mihaescu M, Fuchs L, Papatziamos G, Gutmark E (2013). Planning human upper airway surgery using computational fluid dynamics. Journal of biomechanics.

[25] Mylavarapu G, Murugappan S, Mihaescu M, Kalra M, Khosla S, Gutmark E (2009). Validation of computational fluid dynamics methodology used for human upper airway flow simulations. Journal of biomechanics.

[26] Nithiarasu P, Hassan O, Morgan K, Weatherill N, Fielder C, Whittet H, Ebden P, Lewis K (2008). Steady flow through a realistic human upper airway geometry. International Journal for Numerical Methods in Fluids.

[27] Nouraei S, Nouraei SM, Patel A, Murphy K, Giussani DA, Koury EF, Brown JM, George PJ, Cummins AC, Sandhu GS (2013). Diagnosis of laryngotracheal stenosis from routine pulmonary physiology using the expiratory disproportion index. The Laryngoscope.

[28] Pedley TJ (1977). Pulmonary fluid dynamics. Annual Review of Fluid Mechanics.

[29] Piccinelli M, Veneziani A, Steinman DA, Remuzzi A, Antiga L (2009). A framework for geometric analysis of vascular structures: application to cerebral aneurysms. IEEE transactions on medical imaging.

[30] Pollard A, Uddin M, Shinneeb A-M, Ball C (2012). Recent advances and key challenges in investigations of the flow inside human oro-pharyngeal-laryngeal airway. International Journal of Computational Fluid Dynamics.

[31] Qi S, Li Z, Yue Y, van Triest HJ, Kang Y (2014). Computational fluid dynamics simulation of airflow in the trachea and main bronchi for the subjects with left pulmonary artery sling. Biomedical engineering online.

[32] Reynisson PJ, Scali M, Smistad E, Hofstad EF, Leira HO, Lindseth F, Hernes TAN, Amundsen T, Sorger H, Langø T (2015). Airway segmentation and centerline extraction from thoracic ct–comparison of a new method to state of the art commercialized methods. PloS one.

[33] Rhee JS, Cannon DE, Frank DO, Kimbell JS (2012). Role of virtual surgery in preoperative planning: assessing the individual components of functional nasal airway surgery. Archives of facial plastic surgery.

[34] Stang MT, Armstrong MJ, Ogilvie JB, Yip L, McCoy KL, Faber CN, Carty SE (2012). Positional dyspnea and tracheal compression as indications for goiter resection. Archives of Surgery.

[35] Takeishi N, Miki T, Otani T, Ii S, Morita K, Wada S (2018). Fluid dynamic assessment of tracheal flow in infants with congenital tracheal stenosis before and after surgery. Medical & Biological Engineering & Computing.

[36] Vétel J, Garon A, Pelletier D, Farinas M-I (2008). Asymmetry and transition to turbulence in a smooth axisymmetric constriction. Journal of Fluid Mechanics.

[37] Wootton DM, Sin S, Luo H, Yazdani A, McDonough JM, Wagshul ME, Isasi CR, Arens R (2016). Computational fluid dynamics upper airway effective compliance, critical closing pressure, and obstructive sleep apnea severity in obese adolescent girls. Journal of Applied Physiology.

[38] Xi J, Longest PW, Martonen TB (2008). Effects of the laryngeal jet on nano-and microparticle transport and deposition in an approximate model of the upper tracheobronchial airways. Journal of Applied Physiology.

[39] Zhao K, Malhotra P, Rosen D, Dalton P, Pribitkin EA (2014). Computational fluid dynamics as surgical planning tool: a pilot study on middle turbinate resection. The Anatomical Record.

[40] Zmijanovic Vladeta, Mendez Simon, Moureau Vincent, Nicoud Franck (2016). About the numerical robustness of biomedical benchmark cases: Interlaboratory FDA's idealized medical device. International Journal for Numerical Methods in Biomedical Engineering.

