# Supplementary material for: Assessing Changes in Airflow and Energy Loss in a Progressive Tracheal Compression Before and After Surgical Correction
Source: Ann Biomed Eng. 2019 Dec 2;48(2):822–33. doi: 10.1007/s10439-019-02410-1 (PMC6949211; doi:10.1007/s10439-019-02410-1)
Supplement: Supplementary file 1 — Electronic supplementary material 1 (PDF 3531 kb) [file 10439_2019_2410_MOESM1_ESM.pdf]

Supplementary Information: Assessing changes in airflow and energy loss in a progressive tracheal compression before and after surgical correction.

Qiwei Xiao[1], Raul Cetto[1,2], Denis J. Doorly[1], Alister J. Bates[4],
Jan N. Rose[1], Charlotte McIntyre[1,2], Andrew Comerford[1],
Gitta Madani[3], Neil S. Tolley[2], Robert Schroter[5]

A Convergence Analysis

LES flow computations were performed using the WALE subgrid scale model [3] in Star CCM+, with coefficients C_w = 0.544, K = 0.41. Results for a mesh independence check are shown in table 1. A quasi-DNS simulation employing a 10 million element mesh and 10^{-5} s time step served as a benchmark. Solutions were run with density ρ taken as 1.184 kg.m^{-3}, dynamic viscosity μ as 1.855 $\times$ 10^{-5} N.s.m^{-2}.

	2.2million	3.8million	5.8million	10million
Flow Model	LES	LES	LES	qDNS
Time Step (ms)	0.1	0.05	0.05	0.01
First Wall Layer Thickness (mm)	0.03	0.023	0.014	0.005
Number of Prism Layers	5	5	8	10
Prism Layer Thickness (mm)	0.45	0.36	0.45	0.38
Overall Pressure Loss (Pa)	284.8	275.3	274.5	268.7
Relative Error	5.9%	2.4%	2.1%	0%

Table 1: Mesh convergence check.

The solution parameters for the qDNS simulation are given in table 2. In the table, symbol y^+ is the usual dimensionless normal distance from the wall, $\eta = (\nu^3/\epsilon)^{1/4}$ is the Kolmogorov length scale, (in which ν is kinematic viscosity and ϵ is the dissipation rate at the cell location), V is the volume of a mesh cell, $\tau_\eta = (\nu/\epsilon)^{1/2}$ is the Kolmogorov timescale. Zone H refers to the portion of the computational domain downstream of the constriction, where the turbulence is most intense and consequently the most difficult to resolve. The computation is termed quasi-DNS since although sufficiently fine near the wall, the mesh resolution is not everywhere below the Kolmogorov length scale. As it is known that only a small proportion of the spectral energy content is contained in the wavelength range below 10η, the resolution is considered sufficient for accurate loss estimation.

mean y$^+$	0.19
maximum y$^+$	0.91
mean $\sqrt[3]{V}/\eta$	2.46
max $\sqrt[3]{V}/\eta$<br>% of cells in zone H with $\frac{\sqrt[3]{V}}{\eta} > 8$	9.6<br>0.01 %
mean $\Delta t/\tau_\eta$	0.057

Table 2: qDNS solution parameters.

Table 1 shows that when the mesh resolution increases from 3.8 million to 5.8 million, the reduction of the relative error is modest and both are within 2.5% of the qDNS result, deemed acceptable for loss estimation given other uncertainties associated with geometry definition. The cost of LES simulation is high: 3.8 million elements requiring 2272 core-hours on a HP workstation with two Intel(R) Xeon(R) CPU E5-2630 v3@2.4GHz processors. .

B Turbulence Intensity

Conventionally, calculating turbulence intensity is defined by:

$$TI = \frac{u^{'}}{U} \tag{1}$$

where $u^{'}$ is the root mean square of the velocity fluctuations and U is the mean velocity. Within this study, the mean velocity is defined as the mean velocity at the first trachea ring; for the lowest flow rate (392 ml.s^{-1}) this corresponds to a reference velocity U =4.2 m/s.

C Effects on Wall Shear Stress and Static Pressure

Figure 1 compares the wall shear stress on the back of the trachea of all the T15 geometries. As shown, regions of elevated shear stress correspond both to the constriction and to the impingement of the downstream jet on the trachea wall. There is a marked variation across the various T15 geometries for the shear stress at impingement, with T15 and T15-SG1 corresponding most closely, reflecting the similar breakup of the jet flow in both cases. There is however a pronounced difference in impingement wall shear stress for the simple, truncated geometry (T15-S), compared to all cases incorporating a real or modelled glottis.

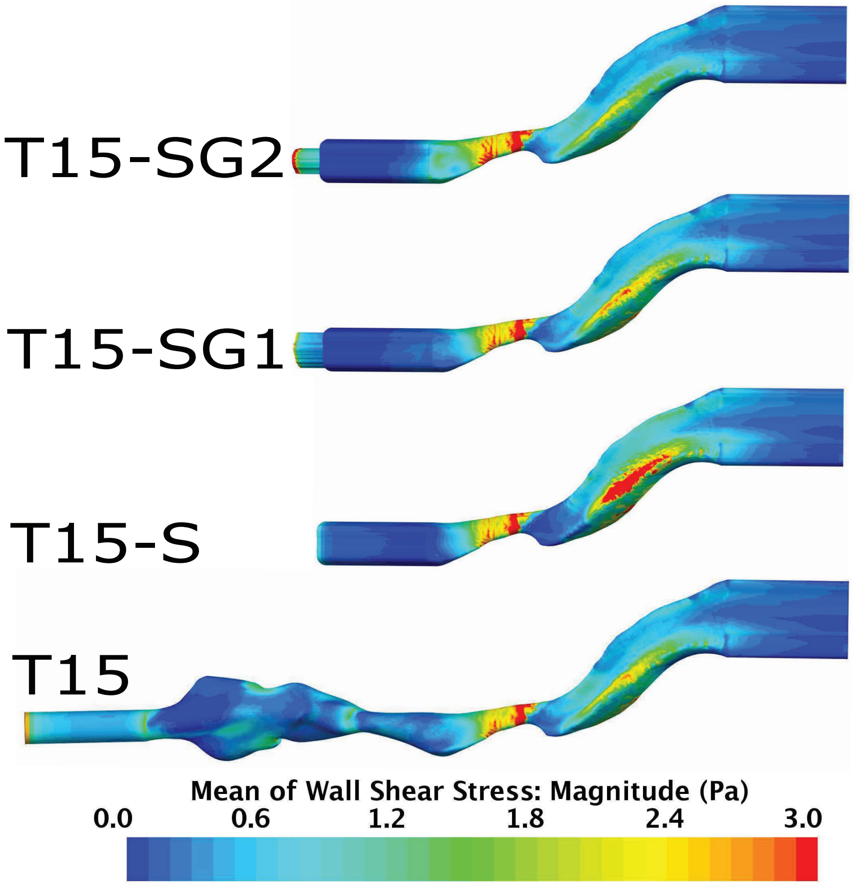

Figure 1: Wall shear stress is shown on the back of trachea surface.

Figure 2 shows the static pressure recovery within various inflow conditions of T15 case,

showing that prolonging the jet breakdown leads to significantly greater differences in the immediate static pressure recovery than was found for total pressure.

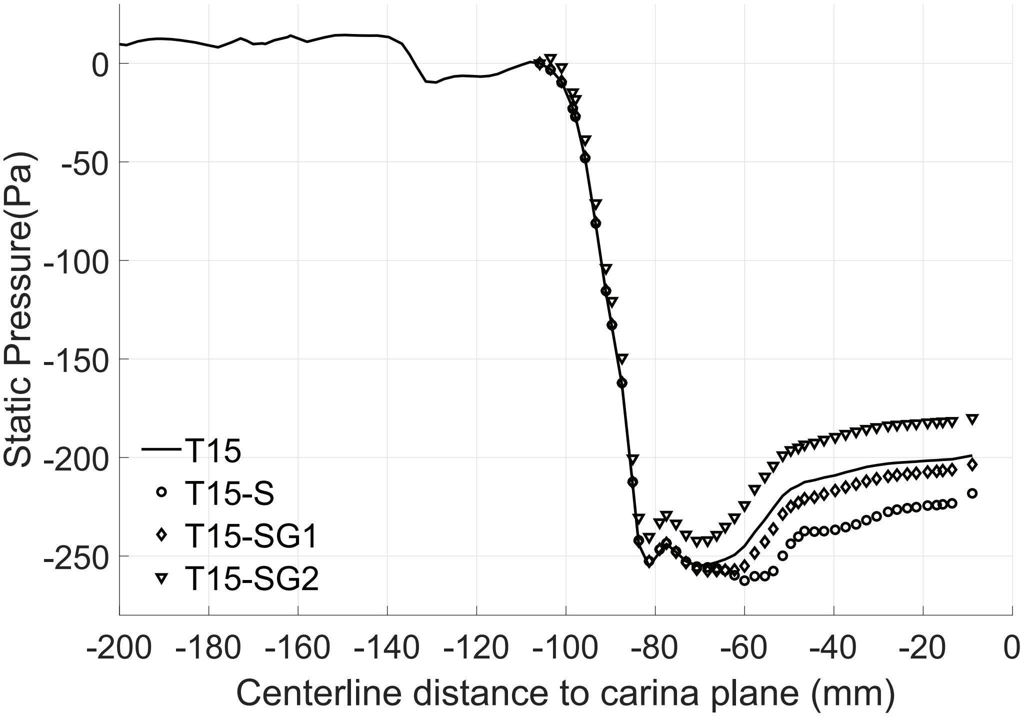

Figure 2: Static pressure distribution of T15 related geometries.

D Inflow disturbance effects on flow loss in T4 geometry

The T4 model was created from scan data that does not include the glottis or supra-glottal airway and features a less severe constriction than at T15, with correspondingly lower flow jet speed. To determine whether the flow effects observed for the T15 geometry would also be manifest in a different constricted airway, a realistic glottis and supraglottal airway was grafted on to the T4 model. This was achieved by slightly inflating the T15 airway to provide a similar first ring area to match that of the T4 geometry.

A comparison of the flow and vorticity renderings for the extended and truncated geometries is shown in figure figure 3

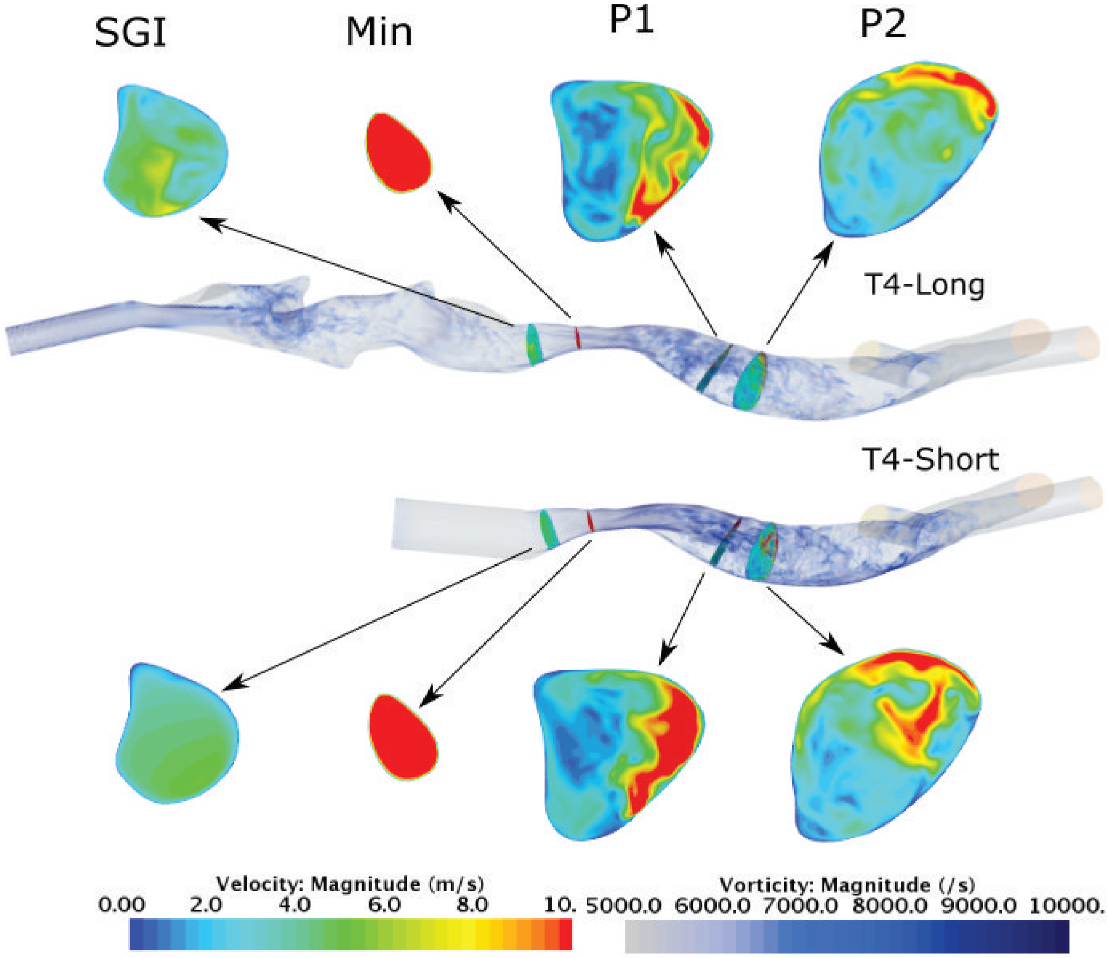

Figure 3: Comparison of flow in T4 geometry with and without addition of a realistic laryngeal airway

As observed for the T15 geometry, inflow disturbances induce an earlier break up of the flow jet emerging from the constriction. Likewise, the total pressure loss measured over the same portion of the trachea at 23.5 l.min^{-1} was 76.1 Pa for the truncated geometry and 71.3 Pa for the extended geometry, showing a comparable relative effect size as found for the T15 geometry.

E Flow loss factors

Traditionally, losses due to restrictions or orifices are estimated using pressure loss factors of the form

$$\Delta P = \frac{1}{2}\rho K \frac{Q^2}{{A_C}^2} = \frac{1}{2}\rho K V_C^2, \qquad (2)$$

where Q is the flow rate, A_C is the contraction area and V_C is the jet velocity at the constriction. For the overall resistance of a manufactured duct, various correlations exist, e.g. ESDU data sheet [1] for localised loss factors, to which are added estimated losses due to pipe friction. Anatomical geometries, being irregular are devoid of neatly defined geometries and are more difficult to characterise. In their study of losses associated with tracheal stenosis Brouns et al[2] found that applying an average value for K of 1.2 gave a good fit to predict losses over the range of constrictions (50% to 90% reduction in tracheal lumen area) studied in their idealised stenotic tracheal geometry. Very similarly, for contraction ratios between 75% and 85%, ref[1] suggests K values in the range 1.01 to 1.19 as appropriate for the longer constriction lengths observed here (length/diameter of narrowest region $>$ 0.8, derived by choosing ESDU parameters λ_b=0.88, and $K^{'}_{0.8}$ in the range 1.15 - 1.35).

F Effect of outflow on energy loss within the trachea

During inhalation, the condition of flow entering the glottis was found to affect losses in flow energy throughout the trachea. Whether conditions at the outflow could have influenced flow in the trachea at some distance upstream is also of interest, since the imaged region may not extend to the carina or lower. For the T4 geometry, we replaced the bifurcation by a simple extrusion of the tracheal cross-section from approximately one diameter above the carina, (creating model T4C). Results for the accumulated loss in energy flux in the original and modified geometry up to this point are plotted in figure 4. As they are virtually indistinguishable we conclude that the loss characteristics within the majority

of the trachea were unaffected by the outflow configuration as expected from experiment[4] and theory[5].

This does not however imply that the outflow condition does not affect the eventual loss due to tracheal constriction. In the paper, it is shown that the peak rate of energy dissipation of the flow jet occurs in the trachea. However the constriction-induced turbulence and flow non-uniformity is not fully dissipated before flow divides at the carina and continues further. There is thus an additional associated loss; however this cannot be predicted without data not merely of the omitted geometry, but the relative flow splits.

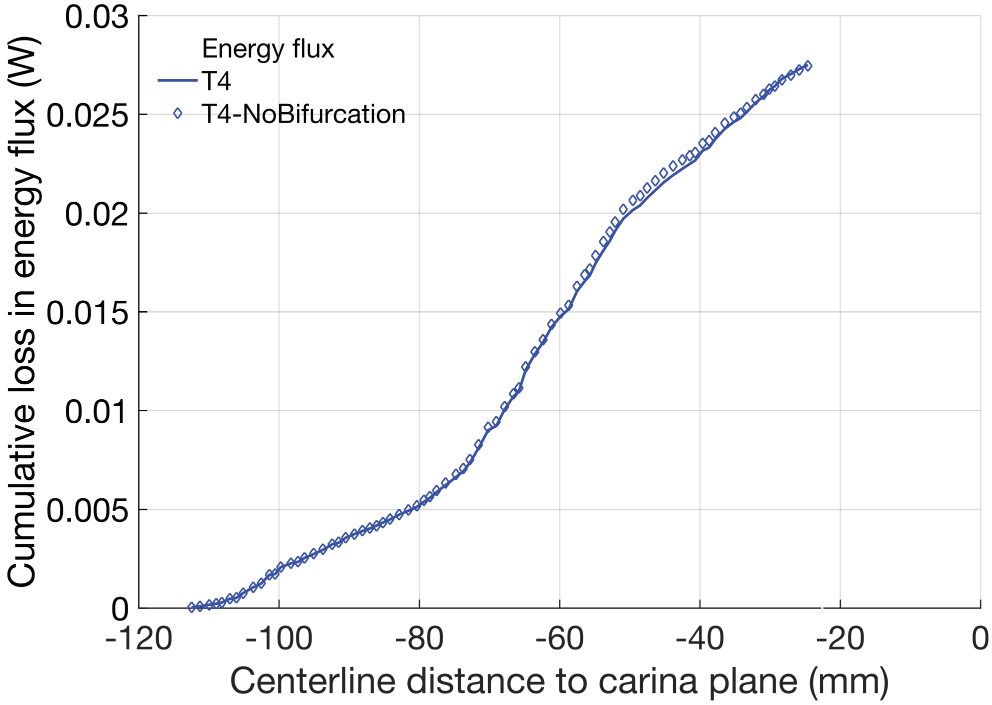

Figure 4: Comparison of accumulated loss in flow energy during inhalation for geometry T4 (includes bifurcation at carina, at 0 mm) and a modified geometry where bifurcation is replaced by extrusion of tracheal cross-section forming a non-bifurcated outflow.

References

1. ESDU 81039 Flow of Liquids: Pressure Losses Across Orifice Plates, Perforated Plates and Thick Orifice Plates in Ducts, ESDU International plc.2009.

2. Brouns, M., S. T. Jayaraju, C. Lacor, J. De Mey, M. Noppen, W. Vincken, and S. Verbanck. Tracheal stenosis: a flow dynamics study. Journal of Applied Physiology 102:1178–1184, 2007.

3. Nicoud, F. and F. Ducros. Subgrid-scale stress modelling based on the square of the velocity gradient tensor. Flow, Turbulence and Combustion 62:183—-200, 1999.

4. Schroter, R. C. and M. Sudlow. Flow patterns in models of human bronchial airways. Resp. Physiol 9:341–355, 1969.

5. Smith, F. T., N. C. Ovenden, P. T. Franke, and D. J. Doorly. What happens to pressure when a flow enters a side branch? Journal of Fluid Mechanics 479:231—-258, 2003.
